# Analyzing water hyacinth plants from two South African rivers for the detection of seven pharmaceuticals and their metabolites

**DOI:** 10.1002/elps.202400101

**Published:** 2024-07-04

**Authors:** Markus Himmelsbach, Franz Mlynek, Wolfgang Buchberger, Lawrence Madikizela, Christian W. Klampfl

**Affiliations:** ^1^ Institute of Analytical and General Chemistry Johannes Kepler University Linz Austria; ^2^ Institute for Nanotechnology and Water Sustainability College of Science Engineering and Technology University of South Africa, Florida Science Campus Johannesburg South Africa

**Keywords:** environmental analysis, metabolization, pharmaceuticals, plant uptake, tramadol

## Abstract

Water hyacinth plants (*Eichhornia crassipes* Mart.) collected from two South African rivers were analyzed in order to investigate their suitability for judging the presence of pharmaceuticals in the water. Thereby, a number of drugs, including amitriptyline, atenolol, citalopram, orphenadrine, lidocaine, telmisartan, and tramadol, could be detected. Particularly for the latter substance, relatively high concentrations (more than 5000 ng g^−1^ dry plant material) were detected in the water plants. Subsequently, the plant extracts were also screened for drug‐derived transformation products, whereby a series of phase‐one metabolites could be tentatively identified.

In 1977, Hignite and Azarnof reported, for the first time, the presence of pharmaceuticals (and drug‐related metabolites) in the aquatic environment [1]. Since then, analyzing environmental waters for pharmaceuticals, their respective metabolites, and later also for personal care products (PPCPs) has become a routine process, controlled by environmental authorities in many countries all over the world. Furthermore, the progress in research within this field is reflected by a large number of scientific papers (for exemplary review papers see Refs. [2, 3, 4, 5, 6, 7]). Main sources for the contamination of environmental waters with these substances are effluents from sewage treatment plants, waste waters from pharmaceutical production plants, and run‐offs from soil where manure from treated animals was applied as a fertilizer. Thereby, concentrations determined in water and soil often allow drawing conclusions to which extent a certain pharmaceutical is used. In African countries, the use (and misuse) of strong painkillers for both humans and livestock is a serious issue [8, 9]. The latter has even led to the (erroneous) claim that the active pharmaceutical ingredient (API) of tramadol (TRM) (a strong painkiller) might also have a natural source being synthesized by a Cameroonian medicinal plant (*Nauclea latifolia*), but subsequent investigations revealed that feces and urine from treated animals were the main reason for the presence of this compound in nature [10]. When analyzing surface waters for the presence of the contaminants discussed, one can, for example, draw water samples, whose analysis resembles the situation in the moment of sampling. An alternative approach would be to monitor the situation over a longer time period, thereby averaging extremely high‐ or low‐concentration values. It is known from a range of research studies that plants grown in a medium containing pharmaceuticals and PPCPs tend to take up these contaminants from the surrounding environment, translocate them to the different plant parts, and, in many cases, even metabolize these compounds (for exemplary reviews see Refs. [11, 12, 13]) For this reason, water plants growing in potentially contaminated surface waters (rivers, lakes) might be a useful tool for evaluating the medium/long‐time exposition of these waters to PPCPs.

Thus, water hyacinth (*Eichhornia crassipes* Mart.) plants were collected from different positions in two South African rivers (Mbokodweni River and Mdloti River)—for a picture of the investigated plant samples, please see Figure S1. Subsequently, these plants were dissected into root, leaves, and stem, freeze‐dried, and finally, the extracts prepared from these materials were analyzed with respect to the presence of drugs and their potential metabolites in the different plant parts. For a more detailed description of the analytical process, please consult the Supporting Information section (Table S1). Screening of the corresponding extracts allowed the detection and quantification of seven APIs, namely, citalopram (CIT), telmisartan, amitriptyline (AMT), orphenadrine (ORP), TRM, atenolol (ATE), and lidocaine. An HPLC‐QTOF‐MS chromatogram for the extract of water hyacinth stem, collected at Mdloti River (collection point 2), can be seen in Figure 1. Quantification of these compounds in the plant extracts was performed using the standard addition method. Table S2 provides an overview of the relevant parameters. As every sample was spiked with a known amount of the drug, it was possible to estimate matrix effects, relevant for further quantitative evaluation. Pronounced, matrix‐related reduction of signal intensities with recoveries ranging from 62% to 69% was observed for AMT, ORP, and CIT, whereas the other four compounds all showed values above 90%. Results from the quantitative analysis of the different parts from the plants collected in the two rivers are provided in Table 1. Thereby, two things caught the attention of the researchers. First, TRM showed much higher concentrations (lying in the µg g^−1^ [dried matter] range) than the other pharmaceuticals. To further confirm these high TRM concentrations, results were checked by employing a different analytical technique (GC–MS), leading to very similar results. These high TRM concentrations point to the widespread use and misuse of this compound in the region. Second, when trying to correlate points of collection with respect to their distance from local WWTPs, no clear trend could be observed. This suggests that other sources for the introduction of TRM must be considered (e.g., the fact that TRM, e.g., is not only used in human medicine but also off‐label use for the treatment of livestock exists [10, 14]) than those via WWTP, which are predominating in European countries.

**FIGURE 1:**
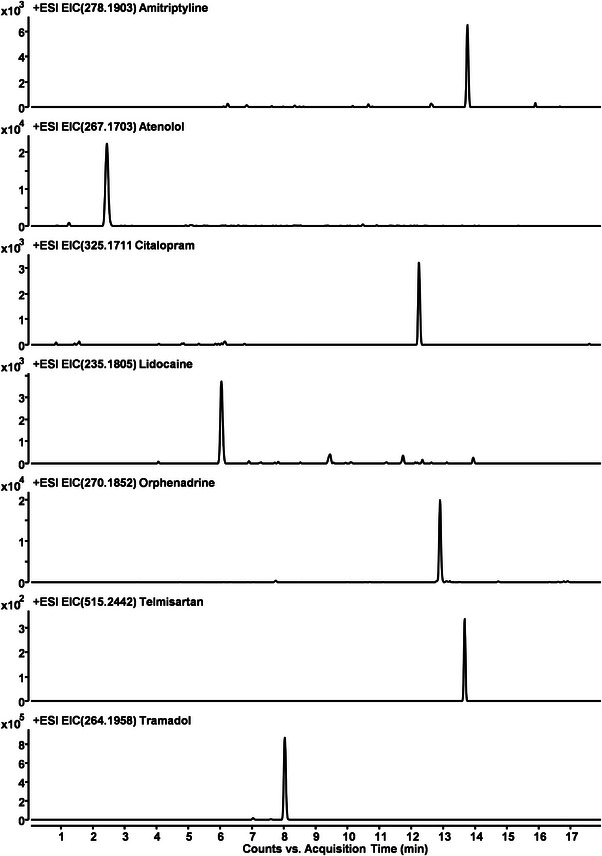
Extracted ion chromatograms for the seven detected parent drugs in extracts (stem) from water hyacinth collected from Mdloti River (collection point 2). For experimental details please, see Supporting Information section.

**TABLE 1:** Concentrations (in ng g^−1^ dry plant material) of the parent drugs in root, stem, and leaves from water hyacinth collected from the Mbokodweni River and from the Mdloti River.

	Mbokodweni River						Mdloti River					
	Collection point 1			Collection point 2			Collection point 1			Collection point 2		
	Roots	Stem	Leaves	Roots	Stem	Leaves	Roots	Stem	Leaves	Roots	Stem	Leaves
Amitriptyline	51	61	10	208	536	162	30	20	14	25	197	48
Atenolol	1230	180	111	239	81	42	344	28	16	982	892	172
Citalopram	6	n.d.	n.d.	81	278	78	22	65	n.d.	14	84	n.d.
Lidocaine	8	50	92	3	29	40	8	125	415	6	90	206
Orphenadrine	137	468	74	132	1285	472	28	142	84	17	413	122
Telmisartan	47	n.d.	n.d.	400	51	26	413	20	25	533	29	n.d.
Tramadol^a^	1263	3102	680	195	2652	1223	319	5456	2320	270	4727	1717
Tramadol^b^	781	2705	833	186	2659	1013	388	5627	2056	277	3653	1356

Abbreviation: n.d., not detected.

^a^ Analyzed by LC–MS/MS.

^b^ Analyzed by GC–MS.

Plants, when brought into contact with PPCPs, can not only take up but also translocate and bio‐transform these substances [15]. For this reason, the presence of metabolites derived from the seven detected APIs in the hyacinth plants was investigated. Search for potential metabolites in the plant extracts was based on procedures published previously [16, 17]. Thereby, when analyzing model plants (garden cress and pea) grown hydroponically, a series of metabolites formed within the plant could be identified tentatively [18, 19, 20]. In the present work, we investigated whether, under the conditions present in the river water, similar metabolites can be found in the hyacinth plants. Thereby, it should be kept in mind that several of these metabolites are also produced by humans or mammals when the parent drug is consumed. So, there may be two different sources for these metabolites as they can either be already present in the water and subsequently be taken up by the plant or formed within the plant from the parent drug. Example of this is *O*‐desmethyltramadol, *N*‐desmethyltramadol, and hydroxytramadol, all metabolites from TRM, which were detected in the hyacinth plants (see Table 2) and can also be found in human urine [21]. For the tentative identification, retention times from HPLC as well as accurate mass measurements and MS/MS data from the QTOF spectra were employed. No metabolites were found for ATE, a fact already reported by Reichl et al. in a study employing garden cress as a model plant [22]. For the other drugs investigated, only phase‐one metabolites were found. As can be seen from Table 2, several transformation products could be tentatively identified in the different parts (root, stem, and leaves) of hyacinth plants collected from Mbokodweni River and Mdloti River.

**TABLE 2:** Drug‐derived metabolites found in the hyacinth plants collected from two South African rivers.

Metabolite	RT (min)	Mbokodweni River						Mdloti River					
		Collection point 1			Collection point 2			Collection point 1			Collection point 2		
		r	S	l	r	s	l	r	s	l	r	s	l
AMT‐OH	10.0	✓	✓	n.d.	✓	✓	✓	n.d.	n.d.	n.d.	n.d.	✓	n.d.
NTP	13.5	n.d.	n.d.	n.d.	✓	✓	✓	n.d.	n.d.	n.d.	n.d.	✓	n.d.
DCP	12.0	n.d.	n.d.	n.d.	✓	✓	✓	✓	✓	n.d.	✓	✓	n.d.
MEGX	4.7	n.d.	n.d.	n.d.	n.d.	n.d.	n.d.	n.d.	✓	✓	n.d.	n.d.	n.d.
DMO	12.6	✓	✓	n.d.	✓	✓	✓	n.d.	✓	✓	n.d.	✓	✓
TEL‐OH	13.5	n.d.	n.d.	n.d.	n.d.	n.d.	n.d.	✓	n.d.	n.d.	✓	n.d.	n.d.
ODT	5.2	✓	✓	✓	✓	✓	✓	✓	✓	✓	✓	✓	✓
NDT	8.2	✓	✓	✓	✓	✓	✓	✓	✓	✓	✓	✓	✓
TRM‐OH	8.7	n.d.	✓	n.d.	n.d.	✓	n.d.	n.d.	✓	n.d.	n.d.	✓	n.d.

Abbreviations: AMT‐OH, 10‐hydroxyamitriptyline; DCP, desmethylcitalopram; DMO, *N*‐desmetylorphenadrine; l, leaves; MEGX, monoethylglycinexylidide; n.d., not detected; NDT, *N*‐desmethyltramadol; NTP, nortriptyline; ODT, *O*‐desmethyltramadol; r, roots; RT, retention time; s, stem; TEL‐OH, hydroxy‐telmisartan; TRM‐OH, hydroxytramadol.

The results from this paper suggest that the analysis of waterborne plants might be suitable for monitoring the presence of emerging contaminants in surface waters on a long‐term basis. Anyhow, additional work, including more controlled growth conditions for the water plants, will be needed to get a clearer picture on the potential of this approach. Furthermore, including a wider range of water plants in the study might provide a deeper insight into variations in uptake, translocation, and metabolization of the contaminants by the different types of plants, allowing us to select the one best suited for monitoring a specific analyte.

## CONFLICT OF INTEREST STATEMENT

The authors declare no conflicts of interest.

## Supporting information

Supporting Information

## Data Availability

The data that support the findings of this study are available from the corresponding author upon reasonable request.
