# Supplementary material for: Analyzing water hyacinth plants from two South African rivers for the detection of seven pharmaceuticals and their metabolites
Source: Electrophoresis. 2024 Jul 4;46(5-6):347–51. doi: 10.1002/elps.202400101 (PMC11952283; doi:10.1002/elps.202400101)
Supplement: Supplementary file 1 — Supporting Information [file ELPS-46--s001.docx]

**ANALYZING WATER HYACINTH PLANTS FROM TWO SOUTH AFRICAN RIVERS FOR THE DETECTION OF SEVEN PHARMACEUTICALS AND THEIR METABOLITES**

Markus Himmelsbach^1^, Franz Mlynek^1^, Wolfgang Buchberger^1^, Lawrence Madikizela^2^ and Christian W. Klampfl^1^*

**Supplementary material**

^
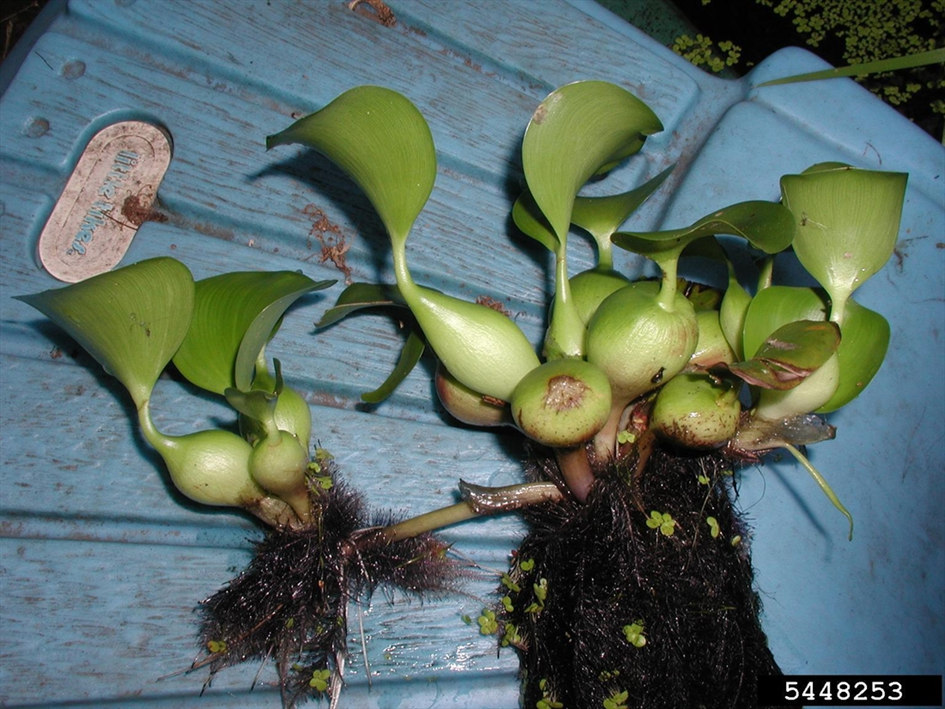
^

Figure S1: Water hyacinth plants as collected from the South African rivers.

**Analysis of plant samples**

300 mg of the freeze dried plant sample were extracted with 5 ml of 50% 0.1 M HCl / 50% MeOH. The samples were vortexed until the plant material was soaked with the extraction media and put into an ultrasonic bath (Elma Ultrasonic, Elmasonic S60 H) for 10 minutes, followed by centrifugation at 4200 g for 16 minutes using a VWR Mega Star 1.6R Centrifuge. The supernatants were separated from the solid residues, filtered through a 0.45 μm nylon filter into an HPLC glass vial and stored at ‑80°C until analysis.

The extracts were analyzed by RP-HPLC using a modular Agilent 1260 HPLC system from Agilent Technologies (Waldbronn, Germany) Separation was performed on a Poroshell 120 EC-C_18_ column (3 x 75 mm, particle size 2.7 µm, Agilent) that was protected with a C_18_ guard column (4 × 3 mm, particle size 3 µm) from Phenomenex (Aschaffenburg, Germany).

A water/acetonitrile gradient was applied. Starting conditions were set to 95% solvent A (water with 0.1% formic acid) and 5% solvent B (acetonitrile with 0.1% formic acid). From minute 0 to 5 solvent B was increased to 15%, from minute 5 to 10 solvent B was increased to 30%, from minute 10 to 15 solvent B was increased to 50%, followed by 5 min 100% solvent B and 5 minutes equilibration with starting conditions, resulting in a total run time of 25 min. The flow rate was set to 0.6 mL min^-1^, the temperature of the column heater was 30 °C and an injection volume of 20 µL was used.

For the targeted analysis of parent drugs in water hyacinth samples, an Agilent 6420 triple quadrupole QqQ-MS/MS (Agilent Technologies, Waldbronn, Germany) equipped with an ESI source was used for detection. The QqQ-MS/MS system was operated in the positive ionization mode. Applied parameters were as follows: capillary voltage 4000 V, drying gas flow rate 11 L min^−1^, drying gas temperature 350 °C, nebulizer pressure 55 psi. The optimized parameters (selected transitions, fragmentor voltages and collision energies) for the multiple reaction monitoring (MRM) mode are given below.

Table S1: Calibration parameters for Amitriptyline, Atenolol, Citalopram, Lidocaine, Orphenadrine, Telmisartan, and Tramadol

| Compound | Calibration range [µg/L] ^a^ | Equation | | R^2^ | average recovery (matrix effect)  over all plant extract samples [%] ^b^ |  |
| --- | --- | --- | --- | --- | --- | --- |
| Amitriptyline | 0.1 - 50 | y = 20507x - 8289.5 | | 0.9996 | 62 |  |
| Atenolol | 0.1 - 100 | y = 6576.9x + 1797.5 | | 0.9993 | 95 |  |
| Citalopram | 0.1 - 25 | y = 40621x - 8174 | | 0.9997 | 66 |  |
| Lidocaine | 0.1 - 50 | y = 88544x - 25160 | | 0.9996 | 91 |  |
| Orphenadrine | 0.1 - 100 | y =155817x - 63813 | | 0.9995 | 69 |  |
| Telmisartan | 0.1 - 50 | y =4896.2x + 3858.9 | | 0.9976 | 105 |  |
| Tramadol | 0.1 - 100 | y = 86053x - 27656 | | 0.9997 | 96 |  |
|  |  | |  |  |  | |

^a^ range was selected to fit the concentrations in the samples

^b^ all extracted plant samples were quantified using standard addition

Table S2: MRM transitions of the APIs

| Compound | Precursor Ion [m/z] | Product Ion [m/z] | Fragmentor (V) | CE (V) | Polarity | Quantifier / Qualifier |
| --- | --- | --- | --- | --- | --- | --- |
| Amitriptyline | 278.2 | 233.1 | 80 | 14 | Positive | Quantifier |
| Amitriptyline | 278.2 | 191.1 | 80 | 25 | Positive | Qualifier |
| Atenlol | 267.2 | 190.1 | 110 | 15 | Positive | Qualifier |
| Atenlol | 267.2 | 145.2 | 110 | 26 | Positive | Quantifier |
| Citalopram | 325.2 | 262.1 | 105 | 17 | Positive | Qualifier |
| Citalopram | 325.2 | 109.1 | 105 | 26 | Positive | Quantifier |
| Lidocaine | 235.2 | 86.1 | 80 | 12 | Positive | Quantifier |
| Lidocaine | 235.2 | 58.1 | 80 | 35 | Positive | Qualifier |
| Orphenadrine | 270.2 | 181.1 | 60 | 10 | Positive | Quantifier |
| Orphenadrine | 270.2 | 166.1 | 60 | 8 | Positive | Qualifier |
| Telmisartan | 515.3 | 497.2 | 80 | 36 | Positive | Quantifier |
| Telmisartan | 515.3 | 276.1 | 80 | 50 | Positive | Qualifier |
| Tramadol | 264.2 | 246.2 | 80 | 3 | Positive | Qualifier |
| Tramadol | 264.2 | 58.1 | 80 | 15 | Positive | Quantifier |

For the tentative identity confirmation of metabolites, the HPLC system was hyphenated with an Agilent 6560 DTIM-QTOF LC-MS/MS equipped with a Dual AJS ESI source (Agilent Technologies, Waldbronn, Germany). The DTIM-QTOF-MS was tuned in the “fragile ion” mode and operated in the positive ionization mode with the following source parameters: drying gas temperature 300 °C, drying gas flow rate 10 L min^-1^, nebulizer pressure 50 psi, sheath gas temperature 300 °C, sheath gas flow rate 10 L min^-1^, capillary voltage 3500 V, nozzle voltage 1000 V and fragmentor 425 V. For MS/MS experiments nitrogen was used as collision gas and collision energies of 20 V and 30 V were applied.
